# ﻿Correlating heatwaves and relative humidity with suicide (fatal intentional self-harm)

**DOI:** 10.1038/s41598-021-01448-3

**Published:** 2021-11-15

**Authors:** Fernando Florido Ngu, Ilan Kelman, Jonathan Chambers, Sonja Ayeb-Karlsson

**Affiliations:** 1 Institute for Risk and Disaster Reduction, University College London, London, UK; 2 Institute for Global Health, University College London, London, UK; 3 University of Agder, Kristiansand, Norway; 4 Institute for Environmental Science, University of Geneva, Geneva, Switzerland; 5 University of Sussex, Brighton, UK; 6 United Nations University’s Institute for Environment and Human Security, Bonn, Germany

**Keywords:** Natural hazards, Risk factors

## Abstract

Empirical evidence suggests that the effects of anthropogenic climate change, and heat in particular, could have a significant impact on mental health. This article investigates the correlation between heatwaves and/or relative humidity and suicide (fatal intentional self-harm) on a global scale. The covariance between heat/humidity and suicide was modelled using a negative binomial Poisson regression with data from 60 countries between 1979–2016. Statistically significant increases and decreases in suicide were found, as well as many cases with no significant correlation. We found that relative humidity showed a more significant correlation with suicide compared to heatwaves and that both younger age groups and women seemed to be more significantly affected by changes in humidity and heatwave counts in comparison with the rest of the population. Further research is needed to provide a larger and more consistent basis for epidemiological studies; to understand better the connections among heat, humidity and mental health; and to explore in more detail which population groups are particularly impacted and why.

## Introduction

Anthropogenic climate change has significant cascading effects on human health globally, including mental health^1–3^. In 2016, 1.1 billion people were estimated to be affected by mental ill health worldwide^4^. Despite its prominence, mental health research and wellbeing support services remain underfunded^5,6^. Climate change influences weather, including by altering heatwaves^1,3^. Temperature changes and rates of change may exceed both humans’ and nature’s ability to adapt^3,7^. Generally, hazards become disasters as a function of vulnerability factors within the population^8,9^. However, heatwaves are notable in that they are both directly exacerbated by climate change along with other factors such as urban heat islands^10,11^ and may create heat and humidity regimes (irrespective of vulnerability factors) that are not habitable for humans^1,7^. Heatwave counts are therefore adopted in the present work as a robust indicator of climate change.

Yet no consensus exists for heatwave definitions^12^. This work adopts the definition of the count of heatwave days per year, where a heatwave is a period of four or more days where the minimum daily temperature is greater than the 99th percentile of local climatology^13^. Relative humidity, which is presented as a percentage to express the proportion of water vapour present in the air, is included since high humidity is known to have detrimental impacts on health^7^. The impacts of heatwaves on physiological and physical health are well-studied^14–16^. Fewer research investigations focus on the implications of heatwaves on mental health and wellbeing.

Proposing a consistent indicator for quantifying mental health across diverse regions is challenging, as is quantifying a person’s state of mind, due to significant cultural differences and perceptions around mental health and illness. This is further complicated by cultural stigma around mental ill health, different traditions and approaches to treatment and healing, and the underreporting of data^17,18^. Datasets are therefore difficult to collect on a large scale. The development of one psychological condition often overlaps with others^19,20^ and diagnoses can be inaccurate, constraining health systems providing appropriate treatment^21,22^. Nevertheless, one indicator used for mental ill health is suicide, related to intentional self-harm as per the categorisation in ICD10 (X60–X84)^23^.

The terminology can be troublesome. From ICD10, completed suicide might be considered fatal intentional self-harm, but this phrase yields an unfortunate acronym and the two are not necessarily exactly the same. Meanwhile, the term “suicide” has deep-seated moral and negative stereotypes, whereas “fatal intentional self-harm” is somewhat distanced from societal stigma and is clear in its definition—assuming that intentionality can be established, which is not always the case. This article selects the term “suicide” since it is most commonly used, while recognising ICD10’s approach and accepting that neither “suicide” nor “fatal intentional self-harm” is entirely satisfactory.

Suicide often occurs under the influence of mental ill health, as research indicates that 88–98% of subjects had a psychiatric diagnosis at the time of death^24–26^. Current studies indicate that the effects of climate change have significant detrimental consequences on mental health^2,27–29^. This science can be broadly summarised around five narratives, which overlap and are not always clearly delineated. First, around the biochemical reaction that the body has in response to extreme heat, resulting in heat stress and the build-up of compound stress over time^29,30^. Second, around medications that counteract mental health diagnoses but inhibit the body’s ability to effectively thermoregulate^28,30^. This results in heat stress and/or the exacerbation of certain mental health conditions, classified as depressive/bipolar ‘disorders’, schizophrenia and other psychoses, dementia and developmental ‘disorders’ including autism^31^. Third, around the impact that heatwaves have as a hazard, which has implications for triggering mental ill health conditions such as post-traumatic stress disorder (PTSD), anxiety and depression^32,33^. Fourth, secondary cascading effects that heatwaves can have on social factors such as societal networks and livelihoods can subsequently affect individual mental health^2,27,34–36^. Fifth, some scholars suggest that ‘climate anxiety’ links to mental ill health through manifestations such as “solastalgia” (distress related to environmental change)^37,38^ and “ecoanxiety”, “ecodepression” and “ecoanger” (worrying about the environment)^39,40^.

The research focus in this article is to investigate whether there is a statistically significant correlation between either heatwaves and/or relative humidity, and suicide on a global scale. This is the first study of its kind and is essential for analysing climate change’s possible impact on mental health. While correlations uncovered do not directly imply causation, this indicator supports understanding and analysis of the effects that heat-humidity combinations might have on mental health. This aids further research while deepening understandings of connections between climate change and mental health.

## Results

Positive and negative significant correlations were found across the varying zero binomial regression modelling results. The incidence risk ratio (IRR) values are presented in this section which indicate the percentage change in suicide as a result of each unit increase of heatwave occurrence or relative humidity. An IRR value of 1 indicates no dependence of suicide on the independent variable, while changes from 1 indicate a corresponding increase/decrease in suicide as a function of the independence variable.

The change in suicide with respect to relative humidity was found to be statistically significant in more instances than the change in suicide with respect to heatwaves. However, there were similar numbers of statistically significant decreases and increases in suicide rates. More instances of significance were found within younger age groups compared to the rest of the population. The results are summarised here with the full results in the supplementary material.

### Suicide rate change significance with respect to heatwave counts

**Figure 1:**
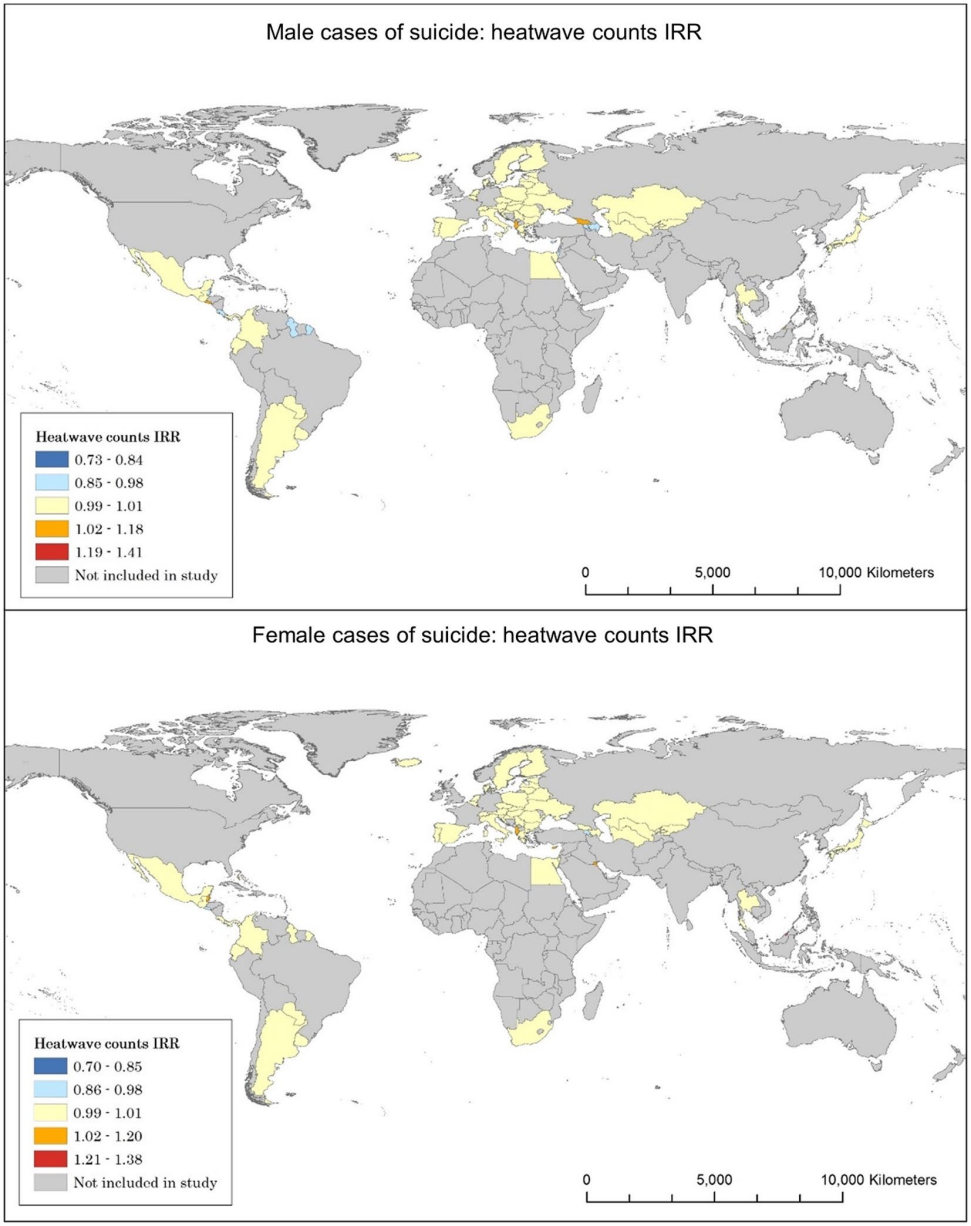
Heatwave and suicide correlations for males and females.

The results summarise suicide stratified by gender across countries (Table 1, Fig. 1). These values indicate that in the countries with a significant result, an increase in suicide of 3.5% is observed for every unit increase of heatwave counts. When the overall figures are stratified by gender, three times as many countries observe a significant increase in suicide in females compared to suicide in males.

**Table 1:** Statistically significant suicide and IRR change with respect to heatwave counts on overall and gendered suicide.

	Overall suicide	Female suicide	Male suicide
Number of countries with a significant suicide-IRR correlation	8	11	7
Average IRR	1.035	1.037	1.016
Number of countries showing a significant increase in suicide for each unit increase of heatwave counts	3	6	2
Average significant increase IRR	1.207	1.133	1.225
Number of countries showing a significant decrease in suicide for each unit increase of heatwave counts	5	5	5
Average significant decrease IRR	0.932	0.922	0.932

There are significantly higher counts for suicide in males than in females. Therefore, the increased significance counts for suicide in females relative to the overall results could indicate that there is a more significant effect among the female population relative to males, and that this effect is masked when considering aggregate suicide values. This highlights the importance of analysing the data by gender stratification.

When stratified by both age and gender, the female age brackets almost all have equal or greater counts of significance compared with the corresponding male age bracket (Table 2). The impact on gendered suicide per unit increase of heatwave counts ranges from − 6.1 to + 5% in suicide for males, and − 6 to + 6.8% in suicide for females.

**Table 2:** Gender and age relevance for suicide and IRR for heatwaves.

Gender—Age bracket	N countries with increase in suicide	Average increase IRR	N countries with decrease in suicide	Average decrease IRR
Male 5–14	0	N/A	10	0.939
Female 5–14	6	1.068	10	0.940
Male 15–24	3	1.023	5	0.970
Female 15–24	3	1.027	6	0.965
Male 25–34	3	1.050	3	0.980
Female 25–34	4	1.018	5	0.978
Male 35–54	1	1.010	2	0.990
Female 35–54	0	N/A	3	0.987
Male 55–74	1	1.010	4	0.988
Female 55–74	1	1.010	5	0.964
Male 75 plus	1	1.200	3	0.989
Female 75 plus	4	1.063	3	0.963

Statistically significant decreases are more common than increases for both male and female age groups. Notably, more counts of significance are observed in younger age groups. This analysis highlights that substantial detail is lost in aggregation which is shown when comparing the stratified data to the overall counts of significance.

### Suicide rate change significance with respect to relative humidity

**Figure 2:**
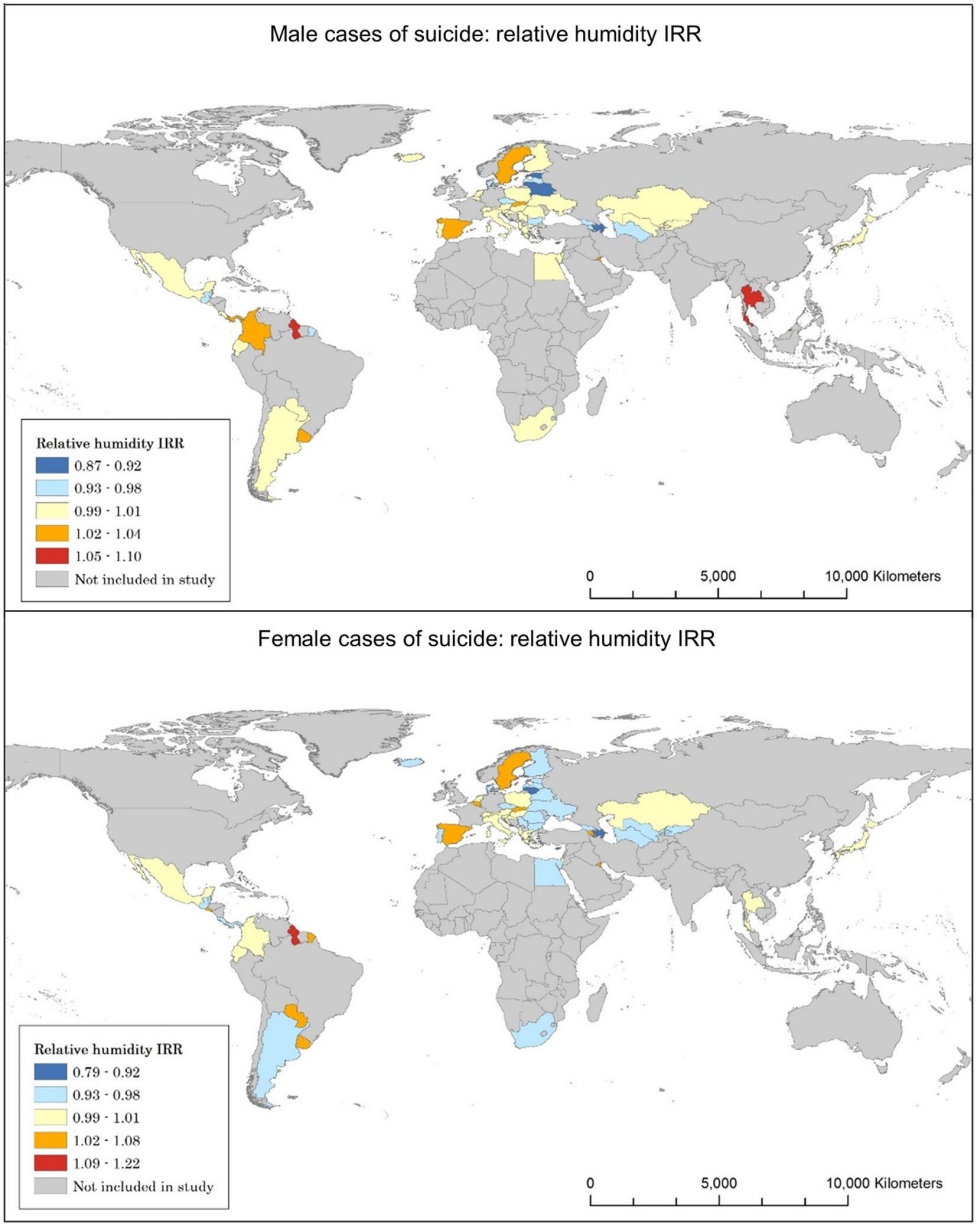
Humidity and suicide correlations for males and females.

Around half of the countries analysed show a significant change in suicide with respect to relative humidity (Tables 3, 4). The impact on overall suicide per unit increase of relative humidity ranges from − 6 to + 4.9%. The average results of countries that show a significant increase in male suicide show a rise in 4.3%, compared to 5.3% in female suicide per unit increase of relative humidity. Countries showing a significant decrease show a decline in suicide mortality by 6.6% for males and 6.1% for females (Fig. 2).

**Table 3:** Statistical significance of relative humidity on overall and gendered suicide.

	Overall suicide	Male suicide	Female suicide
Number of countries with a significant suicide-IRR correlation	28	26	33
Average IRR	0.987	0.984	0.995
Number of countries showing a significant increase in suicide for each unit increase of relative humidity	12	12	16
Average significant increase IRR	1.049	1.043	1.053
Number of countries showing a significant decrease in suicide for each unit increase of relative humidity	16	14	17
Average significant decrease IRR	0.94	0.934	0.939

**Table 4:** Gender and age relevance for suicide and IRR for humidity.

Gender—age bracket	N countries with increase in suicide	Average increase IRR	N countries with decrease in suicide	Average decrease IRR
Male 5–14	16	1.151	17	0.896
Female 5–14	12	1.193	25	0.873
Male 15–24	12	1.073	12	0.930
Female 15–24	13	1.080	15	0.943
Male 25–34	16	1.040	12	0.954
Female 25–34	13	1.054	13	0.946
Male 35–54	12	1.035	14	0.943
Female 35–54	13	1.035	16	0.943
Male 55–74	15	1.035	16	0.935
Female 55–74	13	1.052	16	0.948
Male 75 plus	21	1.071	11	0.946
Female 75 plus	8	1.054	18	0.920

The majority of countries included in the study display a significant relationship between increasing relative humidity and changes in rates of suicide within the 5–14 age bracket, in addition to the male age brackets of those 55 and above. Notably, the range of change is much broader than the figures that are shown for the overall data, with data showing decreases of 12.7% to increases of 19.3%. Total counts of significance throughout the age groups take the shape of an inverted bell curve, falling after the 5–14 bracket and rising above age 55, indicating that those in the youngest and eldest age groups will experience the most change should relative humidity increase.

## Discussion

Previous studies suggest a positive correlation between heatwaves, humidity and suicide^2,27,28^. By contrast, this study found statistically significant increases and decreases in suicide with respect to increases in heatwave counts and relative humidity, suggesting more mixed influence. Being a macro level study, it is possible that some of these correlations found here are spurious or influenced by other factors. Being the first study of its kind, it is clear that further work is needed to understand these correlations and associated IRR changes.

The results highlight the complexity of factors that influence mental health and wellbeing. As many countries repeatedly showed an IRR divergence (with 95% confidence), there is likely a relationship between both heatwave occurrences and relative humidity with suicide. The nature of this relationship including possibilities for causality are clouded by the array of other potential influencing factors. The results indicate that relative humidity consistently has greater counts of significant relationships with rates of suicide compared to heatwaves. Additionally, relative humidity and counts of heatwaves do not depend on one another in their relationship with suicide. Countries where rates of suicide significantly increased or decreased as a result of one of these variables were often distinct.

A few interesting patterns in the results stand out. Female cases of suicide are more significantly affected by changes in the independent variables. This was true both in the degree of significance observed across the age groups and in the number of countries where significance was observed. This result could be explained by some gender trends such as that women more often are diagnosed with mental health and wellbeing issues than men^41,42^.

Such gendered diagnoses feed into a long history of women being assumed to be overemotional, hysterical and therefore needing to be controlled^43^. Historically, scientific research is heavily framed around a bias of gendered binary opposites^42,44^. Women are also more likely to be prescribed psychotropic drugs than men across every adult age category^42,45,46^. As noted, the link between psychotropic medications and the failure of thermoregulatory processes could therefore make women more vulnerable to the effects of heatwaves. Past work also indicates that existing social inequality, inequity, marginalisation and discrimination make women more vulnerable to climatic and other environmental impacts in general. For example, women tend to eat less during periods of food insecurity, are ostracised during menstruation in many places, are often carers and so put themselves in danger to help others, face extensive physical and sexual violence, harassment and abuse during evacuation as well as in shelters and suffer more post-disaster ill health^47–50^.

Younger age groups (in both ages 5–14 and 15–24) exhibited more counts of significance than those in any other age group in the analysis. This might be explained by the fact that the brain and nervous system are still developing at younger ages and are more susceptible to the influence of environmental changes^51,52^. Furthermore, children experience some similar social vulnerabilities to women, in terms of patterns of mortality, abuse and violence, and their growth and development are likely to be impeded during periods of food insecurity^53–56^.

Future studies should examine different metrics for atmospheric conditions related to heatwaves, such as wet-bulb globe temperature or humidex to provide more specific information regarding the relationship between these environmental factors and wellbeing. Bioclimatic temperature indicators could be used, such as the Universal Thermal Climate Index^57^ aiming to measure the effective physiological impact of the combination of temperature, humidity and insolation amongst others.

This study considered only the direct correlation between climate conditions and suicide within a given year. Since mental health impacts may take time to develop, future work could consider lag times of one to eight years, to examined chronic relationships between heatwaves and mental health within countries. Further data collection or gap-filling could be used to extend the time series available for analyses, which should be able to more reliably reveal trends. To counter the issue of aggregation that has influenced the results, an approach which focuses on regions within each country would provide more nuanced and accurate data when compared to countrywide analysis, as long as the variables were available at the required resolutions.

## Conclusion

The results of this investigation showed both significant increases and decreases in country rates of suicide. Relative humidity was shown to be more significantly related to suicide than heatwave counts. Younger people and women appeared to be more affected than other population groups. These findings are important for policy formulation in terms of being aware that patterns of mental health impacts and responses to increasing heat and relative humidity are not consistent around the world, so more localised understandings and responses are needed, which might need to factor in cultural differences. Any interventions should prioritise the groups most affected, but without neglecting others whose vulnerability otherwise may increase, especially seeking to overcome stigmas.

Further research ought to investigate the mixture of increases and decreases observed in the results. The diverse factors surrounding health and wellbeing are most likely changing and will change further, with both societal and environmental influences. The degree and type of change depends on various elements that must be fully identified and further researched, especially with regards to the five narratives of climate change impacting mental health and wellbeing. This includes confounders (such as other weather and demographic variables), factors with observational limitations (such as intentionality of self-harm), changing baselines (such as for diagnoses and prescriptions) and data limitations, all of which contribute to explaining the wide variation of results.

Continuing gender-based and age-based approaches helps in tackling the harmful effects that climate change has on mental health and wellbeing. Many other individual vulnerability factors were not investigated and need to be considered, such as socioeconomic status, disability and sexuality, alongside societal vulnerability factors, such as health systems, access to healthcare and mental health stigmas. While further investigation is necessary to better establish the fundamental causes behind the mixed relationship uncovered in this work, this research provides a solid foundation and insight into the effects that climate change has had, and is likely to continue to have, on mental health and wellbeing.

## Methods

Data for heatwave occurrences (independent variable), relative humidity (confounding variable), population (offset variable) and suicide (dependent variable) are analysed using a negative binomial model across sixty countries for the years 1979–2016 (see the ‘data availability statement’).

A Poisson model is selected as the mode of analysis as the data are count integers; since the data are over-dispersed, a negative binomial regression model is chosen over a standard Poisson regression^58^. As Poisson modelling does not provide results when “N/A” values are present, any years that contained these values were removed entirely. Finally, any countries that failed to include complete data for at least two thirds of the total timeframe (at least 25 years from 1979 to 2016) were omitted from this study to exclude any unreliable results produced from incomplete data. Once these requirements were applied, 60 countries were found to have sufficient data.

The negative binomial model is run using different stratifications of suicide by age and gender for each country. The statistical significance of the model fit is calculated, and the upper and lower bounds of confidence interval values are extracted. Furthermore, to summarise results across countries we present the counts of the number of countries where a statistically significant increase or decrease in suicide was found with respect to the given variable, for the suicide stratifications analysed.

The incidence risk ratio (IRR) value is presented, which indicates the percentage change in suicide as a result of each unit increase of heatwave occurrence or relative humidity. An IRR value of 1 indicates no dependence of suicide on the independent variable; changes from 1 indicate a corresponding increase/decrease in suicide as a function of the independence variable. These results are recorded to three decimal places to show accurate changes in suicide: each 0.1% impact on rates of suicide is essential to note, as this equates to thousands of lives annually. Visualisations of the results have been produced using ArcGIS displaying the IRR value for each independent variable per country analysed.

The results showing the IRR figures and values for the countries included for each model run are in the supplementary material. The full code written in R programming language is an open access dataset^59^.

### Data availability and summary

The data sources are all publicly available:

- Suicide and population data^60^ are sourced from the WHO’s mortality database, presenting data by country, year, age, sex and cause of death as reported by countries.
- Heatwaves count data^13^ are based on the heatwave definition adopted of four or more days of temperature that exceed the 99th percentile of temperature applied to ERA5 temperature data for the years 1980–2018.
- Relative humidity data^61^ are integrated Surface Database Humidity land data provided by the UK’s Met. Office Hadley Centre, where relative humidity is extracted.
- World map shapefile^62^ gives raster representation of countries, which the above data is clipped onto for display.

All data analyses performed using human data adhered to relevant ethical guidelines on human data usage.

Important limitations relate to the availability, formats and accuracy of the datasets. Many countries did not provide full sets of data in one of the three variables, which resulted in their exclusion from the study. Many countries with the largest populations were left out, such as the United States of America, China, India and Russia. The selection also led to the overrepresentation of mid- and high-latitude countries. Since the change in heat and humidity is of most relevance to this indicator, this selection bias may be less of an issue than it could have been with an overrepresentation of countries located in warmer and more humid climates. The reason is that countries with a warmer and more humid climate may see the most impacts on mental health and wellbeing in relation to sudden-onset cold weather or temperature drops than due to heat or humidity increases. That is, for suicide, magnitude of temperature and humidity change might be more or as important than/as direction of change, an aspect to be further explored if data were available.

Additionally, populations within countries can show large variations sub-nationally. National comparisons could therefore obscure more local comparisons, especially as confounders such as health data collected and access to health systems can vary substantially within countries.

## Supplementary Information

Supplementary Information.
