# Author Correction: Correlating heatwaves and relative humidity with suicide (fatal intentional self-harm)

**DOI:** 10.1038/s41598-021-03089-y

**Published:** 2021-12-01

**Authors:** Fernando Florido Ngu, Ilan Kelman, Jonathan Chambers, Sonja Ayeb-Karlsson

**Affiliations:** 1 Institute for Risk and Disaster Reduction, University College London, London, UK; 2 Institute for Global Health, University College London, London, UK; 3 University of Agder, Kristiansand, Norway; 4 Institute for Environmental Science, University of Geneva, Geneva, Switzerland; 5 University of Sussex, Brighton, UK; 6 United Nations University’s Institute for Environment and Human Security, Bonn, Germany

Correction to: *Scientific Reports* https://doi.org/10.1038/s41598-021-01448-3, published online 15 November 2021

The original version of this Article contained an error in Table 3 where rows 3 and 5 were incorrect,

“Number of countries showing a significant increase in suicide for each unit increase of heatwave counts”

now reads:

“Number of countries showing a significant increase in suicide for each unit increase of relative humidity”

And,

“Number of countries showing a significant decrease in suicide for each unit increase of heatwave counts”

now reads:

“Number of countries showing a significant decrease in suicide for each unit increase of relative humidity”

The original Article has been corrected.

