# Supplementary material for: ﻿Correlating heatwaves and relative humidity with suicide (fatal intentional self-harm)
Source: Sci Rep. 2021 Nov 15;11:22175. doi: 10.1038/s41598-021-01448-3 (PMC8593067; doi:10.1038/s41598-021-01448-3)
Supplement: Supplementary file 1 — Supplementary Information. [file 41598_2021_1448_MOESM1_ESM.docx]

Correlating heatwaves and relative humidity with suicide (fatal intentional self-harm)

Fernando Florido Ngu, Ilan Kelman, Jonathan Chambers, and Sonja Ayeb-Karlsson

# 7 | Supplementary Material

7.1 | Negative binomial results showing complete IRR figures and significance for the impact of heatwaves on suicide.

| Country | Overall heatwave IRR | Overall heatwave significance | Male heatwave IRR | Male heatwave significance | Female heatwave IRR | Female heatwave significance |
| --- | --- | --- | --- | --- | --- | --- |
| Albania | 1.03 | Significant increase | 1.04 | Significant increase | 1.02 | Significant increase |
| Argentina | 1 | Not Significant | 1 | Not Significant | 1 | Not Significant |
| Armenia | 0.99 | Significant decrease | 0.99 | Significant decrease | 0.98 | Significant decrease |
| Austria | 1 | Not Significant | 1 | Not Significant | 1 | Not Significant |
| Azerbaijan | 0.99 | Not Significant | 0.99 | Not Significant | 0.99 | Not Significant |
| Bahamas | 0.97 | Not Significant | 0.96 | Not Significant | 1.02 | Not Significant |
| Belarus | 1 | Not Significant | 1 | Not Significant | 1 | Not Significant |
| Belgium | 1 | Not Significant | 1 | Not Significant | 1 | Not Significant |
| Belize | 0.97 | Significant decrease | 0.96 | Significant decrease | 1.02 | Not Significant |
| Brunei Darussalam | 1.21 | Significant increase | 1.15 | Not Significant | 1.38 | Significant increase |
| Bulgaria | 1 | Not Significant | 1 | Not Significant | 1 | Not Significant |
| Colombia | 1 | Not Significant | 1 | Not Significant | 1 | Not Significant |
| Costa Rica | 0.99 | Significant decrease | 0.99 | Significant decrease | 1 | Not Significant |
| Croatia | 1 | Not Significant | 1 | Not Significant | 1 | Not Significant |
| Cyprus | 1.01 | Not Significant | 0.99 | Not Significant | 1.07 | Significant increase |
| Czech Republic | 1 | Not Significant | 1 | Not Significant | 1 | Not Significant |
| Denmark | 1 | Not Significant | 1 | Not Significant | 1 | Not Significant |
| Ecuador | 1 | Not Significant | 1 | Not Significant | 1 | Not Significant |
| Egypt | 1 | Not Significant | 1 | Not Significant | 1 | Not Significant |
| El Salvador | 0.99 | Not Significant | 1.01 | Not Significant | 0.95 | Significant decrease |
| Estonia | 1 | Not Significant | 1 | Not Significant | 1 | Not Significant |
| Finland | 1 | Not Significant | 1 | Not Significant | 1 | Not Significant |
| French Guiana | 0.99 | Not Significant | 0.99 | Not Significant | 0.99 | Not Significant |
| Georgia | 1 | Not Significant | 1.01 | Not Significant | 1 | Not Significant |
| Greece | 1 | Not Significant | 1 | Not Significant | 1 | Not Significant |
| Guatemala | 1 | Not Significant | 1 | Not Significant | 1 | Not Significant |
| Guyana | 0.99 | Not Significant | 0.99 | Not Significant | 0.99 | Significant decrease |
| Hungary | 1 | Not Significant | 1 | Not Significant | 1 | Not Significant |
| Iceland | 1 | Not Significant | 1 | Not Significant | 0.99 | Not Significant |
| Israel | 0.99 | Significant decrease | 0.99 | Significant decrease | 1 | Not Significant |
| Italy | 1 | Not Significant | 1 | Not Significant | 1 | Not Significant |
| Japan | 1 | Not Significant | 1 | Not Significant | 1 | Not Significant |
| Kazakhstan | 1 | Not Significant | 1 | Not Significant | 1 | Not Significant |
| Kuwait | 1.01 | Not Significant | 1 | Not Significant | 1.03 | Significant increase |
| Kyrgyzstan | 1 | Not Significant | 1 | Not Significant | 1 | Not Significant |
| Latvia | 1 | Not Significant | 1 | Not Significant | 1 | Not Significant |
| Lithuania | 1 | Not Significant | 1 | Not Significant | 1 | Not Significant |
| Luxembourg | 1.38 | Significant increase | 1.41 | Significant increase | 1.29 | Significant increase |
| Mexico | 1 | Not Significant | 1 | Not Significant | 1 | Not Significant |
| Moldova | 1 | Not Significant | 1 | Not Significant | 1 | Not Significant |
| Netherlands | 1 | Not Significant | 1 | Not Significant | 1 | Not Significant |
| Panama | 1 | Not Significant | 1 | Not Significant | 1 | Not Significant |
| Paraguay | 1 | Not Significant | 1 | Not Significant | 1 | Not Significant |
| Poland | 1 | Not Significant | 1 | Not Significant | 1 | Not Significant |
| Portugal | 1 | Not Significant | 1 | Not Significant | 1 | Not Significant |
| Romania | 1 | Not Significant | 1 | Not Significant | 1 | Not Significant |
| Russian Federation | 1 | Not Significant | 1 | Not Significant | 1 | Not Significant |
| Serbia | 1 | Not Significant | 1 | Not Significant | 1.01 | Significant increase |
| Slovakia | 1 | Not Significant | 1 | Not Significant | 1 | Not Significant |
| Slovenia | 1 | Not Significant | 1 | Not Significant | 0.99 | Significant decrease |
| South Africa | 1 | Not Significant | 1 | Not Significant | 1 | Not Significant |
| Spain | 1 | Not Significant | 1 | Not Significant | 1 | Not Significant |
| Sweden | 1 | Not Significant | 1 | Not Significant | 1 | Not Significant |
| Switzerland | 1 | Not Significant | 1 | Not Significant | 1 | Not Significant |
| Thailand | 1 | Not Significant | 1 | Not Significant | 1 | Not Significant |
| Trinidad and Tobago | 0.72 | Significant decrease | 0.73 | Significant decrease | 0.7 | Significant decrease |
| Turkmenistan | 1 | Not Significant | 1 | Not Significant | 1 | Not Significant |
| Ukraine | 1 | Not Significant | 1 | Not Significant | 1 | Not Significant |
| Uruguay | 1 | Not Significant | 1 | Not Significant | 1 | Not Significant |
| Uzbekistan | 1 | Not Significant | 1 | Not Significant | 1 | Not Significant |

7.2 | Results showing the IRR values and significance of heatwaves on male suicide for each age group.

## 7.3 | Results showing the IRR values and significance of heatwaves on female suicide for each age group.

## 7.4 | Negative binomial results showing complete IRR figures and significance for the impact of humidity on suicide.

| Country | Overall humidity IRR | Overall humidity significance | Male humidity IRR | Male humidity significance | Female humidity IRR | Female humidity significance |
| --- | --- | --- | --- | --- | --- | --- |
| Albania | 1 | Not Significant | 1 | Not Significant | 1 | Not Significant |
| Argentina | 1 | Not Significant | 1 | Not Significant | 0.99 | Not Significant |
| Armenia | 0.98 | Significant decrease | 0.96 | Significant decrease | 1.03 | Significant increase |
| Austria | 1.01 | Not Significant | 1.01 | Not Significant | 1.01 | Not Significant |
| Azerbaijan | 0.91 | Significant decrease | 0.92 | Significant decrease | 0.87 | Significant decrease |
| Bahamas | 0.91 | Significant decrease | 0.94 | Significant decrease | 0.79 | Significant decrease |
| Belarus | 0.93 | Significant decrease | 0.92 | Significant decrease | 0.96 | Significant decrease |
| Belgium | 1.01 | Significant increase | 1.01 | Significant increase | 1.02 | Significant increase |
| Belize | 0.93 | Significant decrease | 0.93 | Significant decrease | 0.94 | Not Significant |
| Brunei Darussalam | 1.09 | Significant increase | 1.05 | Not Significant | 1.22 | Significant increase |
| Bulgaria | 0.98 | Significant decrease | 0.98 | Significant decrease | 0.98 | Significant decrease |
| Colombia | 1.01 | Not Significant | 1.02 | Not Significant | 1 | Not Significant |
| Costa Rica | 1 | Not Significant | 1.01 | Not Significant | 0.97 | Significant decrease |
| Croatia | 0.99 | Not Significant | 0.99 | Not Significant | 1 | Not Significant |
| Cyprus | 0.87 | Significant decrease | 0.88 | Significant decrease | 0.86 | Significant decrease |
| Czech Republic | 0.98 | Significant decrease | 0.98 | Significant decrease | 0.98 | Significant decrease |
| Denmark | 0.98 | Significant decrease | 0.98 | Significant decrease | 0.95 | Significant decrease |
| Ecuador | 1.01 | Not Significant | 1 | Not Significant | 1.01 | Not Significant |
| Egypt | 0.99 | Not Significant | 0.99 | Not Significant | 0.98 | Not Significant |
| El Salvador | 1.01 | Not Significant | 1 | Not Significant | 1.03 | Significant increase |
| Estonia | 0.9 | Significant decrease | 0.88 | Significant decrease | 0.96 | Significant decrease |
| Finland | 0.99 | Significant decrease | 0.99 | Not Significant | 0.99 | Significant decrease |
| French Guiana | 0.98 | Not Significant | 0.95 | Not Significant | 1.08 | Not Significant |
| Georgia | 0.95 | Significant decrease | 0.96 | Significant decrease | 0.93 | Significant decrease |
| Greece | 1 | Not Significant | 1 | Not Significant | 1 | Not Significant |
| Guatemala | 0.94 | Significant decrease | 0.94 | Significant decrease | 0.96 | Significant decrease |
| Guyana | 1.1 | Significant increase | 1.08 | Significant increase | 1.11 | Significant increase |
| Hungary | 1 | Not Significant | 1 | Not Significant | 1 | Not Significant |
| Iceland | 0.98 | Not Significant | 0.99 | Not Significant | 0.98 | Not Significant |
| Israel | 1.01 | Not Significant | 1.01 | Significant increase | 1 | Not Significant |
| Italy | 1.01 | Not Significant | 1.01 | Not Significant | 1.01 | Not Significant |
| Japan | 1 | Not Significant | 1 | Not Significant | 1.01 | Significant increase |
| Kazakhstan | 1 | Not Significant | 1 | Not Significant | 1 | Not Significant |
| Kuwait | 1.03 | Significant increase | 1.02 | Significant increase | 1.06 | Significant increase |
| Kyrgyzstan | 1.01 | Not Significant | 1.01 | Not Significant | 0.99 | Not Significant |
| Latvia | 0.93 | Significant decrease | 0.93 | Significant decrease | 0.95 | Significant decrease |
| Lithuania | 0.88 | Significant decrease | 0.87 | Significant decrease | 0.9 | Significant decrease |
| Luxembourg | 1.01 | Not Significant | 1.01 | Not Significant | 1.03 | Significant increase |
| Mexico | 1 | Not Significant | 1 | Not Significant | 1.01 | Not Significant |
| Moldova | 1.01 | Significant increase | 1.01 | Not Significant | 1.02 | Significant increase |
| Netherlands | 1 | Not Significant | 1 | Not Significant | 1 | Not Significant |
| Panama | 1.02 | Not Significant | 1.02 | Not Significant | 0.99 | Not Significant |
| Paraguay | 1.01 | Not Significant | 1 | Not Significant | 1.02 | Significant increase |
| Poland | 1.01 | Not Significant | 1.01 | Significant increase | 1 | Not Significant |
| Portugal | 0.99 | Not Significant | 0.99 | Not Significant | 0.99 | Not Significant |
| Romania | 0.99 | Not Significant | 0.99 | Not Significant | 0.99 | Not Significant |
| Russian Federation | 1.1 | Significant increase | 1.11 | Significant increase | 1.07 | Significant increase |
| Serbia | 0.99 | Not Significant | 1 | Not Significant | 0.99 | Significant decrease |
| Slovakia | 1.03 | Significant increase | 1.03 | Significant increase | 1.04 | Significant increase |
| Slovenia | 1 | Not Significant | 1 | Not Significant | 1 | Not Significant |
| South Africa | 0.99 | Not Significant | 1 | Not Significant | 0.98 | Significant decrease |
| Spain | 1.03 | Significant increase | 1.03 | Significant increase | 1.03 | Significant increase |
| Sweden | 1.02 | Significant increase | 1.02 | Significant increase | 1.03 | Significant increase |
| Switzerland | 1 | Not Significant | 1.01 | Not Significant | 1 | Not Significant |
| Thailand | 1.04 | Significant increase | 1.06 | Significant increase | 1.01 | Not Significant |
| Trinidad and Tobago | 1.1 | Significant increase | 1.1 | Significant increase | 1.1 | Significant increase |
| Turkmenistan | 0.98 | Not Significant | 0.98 | Not Significant | 0.99 | Not Significant |
| Ukraine | 0.99 | Not Significant | 0.99 | Not Significant | 0.99 | Not Significant |
| Uruguay | 1.03 | Significant increase | 1.04 | Significant increase | 1.03 | Significant increase |
| Uzbekistan | 0.98 | Significant decrease | 1 | Not Significant | 0.95 | Significant decrease |

## 7.5 | Results showing the IRR values and significance of relative humidity on male suicide for each age group.

## 7.6 | Results showing the IRR values and significance of relative humidity on female suicide for each age group.
